# Challenges in the management of the child with Duchenne muscular dystrophy in a resource poor setting:a case report

**DOI:** 10.11604/pamj.2014.19.227.3137

**Published:** 2014-10-30

**Authors:** Kelechi Kenneth Odinaka, Emeka Charles Nwolisa

**Affiliations:** 1 Department of Paediatrics, Federal Medical Centre Owerri, Nigeria; 2 Department of Paediatrics Federal Medical Centre Owerri, Nigeria

**Keywords:** Duchenne muscular dystrophy, genetic, paediatricians

## Abstract

Duchenne muscular dystrophy is a progressive genetic disease with no cure at present. Children suffering from this disease eventually become wheelchair bound and die in their late teens. Paediatricians caring for the child with Duchenne Muscular Dystrophy in resource poor settings face a lot challenges. These challenges include: poverty, inadequate multidisciplinary care, emotional burn-out of parents and lack of facilities for dystrophin assay or genetic testing.

## Introduction

Duchenne muscular dystrophy (DMD) is an X-linked recessive genetic disease which affects all races and ethnic groups with an incidence of 1 in 3500 male live births [1, 2]. The disease arises as a result of lack of a skeletal muscle protein called dystrophin. Although the disease is inherited as X-linked recessive disorder, about thirty percent of cases are due to spontaneous mutations [3].

Affected individuals can have mildly delayed motor milestones and most are unable to run and jump properly due to proximal muscle weakness, which also results in the use of the classic Gowers’ manoeuvre when arising from the floor [4]. Most patients are diagnosed at approximately 5 years of age, when their physical ability differs obviously from that of their peers [4]. Progression of muscle weakness and leg contractures leads to loss of walking and complete wheelchair dependence at a mean age of 9.5 years [5]. Majority of patients suffering from DMD if untreated, die in their late teens or early twenties mainly from respiratory insufficiency, although 20% of deaths are from dilated cardiomyopathy [5, 6].

Although advances have been made in the management of DMD over the last two decades that have altered the natural history of the disease, with most patients surviving into adulthood; there is still no cure for the disease at present. This places a huge burden on Paediatricians and families caring for children with DMD in resource poor-settings.

We report a case of DMD to highlight some challenges Paediatricians encounter when managing a child with DMD in a resource-poor setting.

## Patient and observation

Master E.C, an 8year old male, presented at the children outpatient clinic of Federal Medical Centre owerri, Imo State, with a 5 year history of difficulty getting up from a sitting position, toe walking and progressive weakness of the limbs.

Prior to presentation at Federal Medical Centre (FMC) owerri, parents had taken the child to several patent medicine shops and traditional healers, but his condition did not improve, thus necessitating the presentation to our facility for treatment.

He is the second child in a family with five children (all males). There was no history of similar illness among siblings. He is in Nursery 3 and performs poorly in school. Parents have primary school level of education. His mother sells crayfish and the father is a truck pusher.

On examination, he was withdrawn and had a waddling gait. He had hypertrophy of the calf muscles and Gower's sign was positive. His blood pressure was 90/60mmHg. The other systems were essentially normal. Laboratory investigations revealed a normal Haemoglobin estimation. Urinalysis, chest X-ray and electrocardiogram were all normal. Serum creatinine kinase was 3863 iµl (normal 25 iµ/l). Muscle biopsy was not done.

A clinical diagnosis of DMD was made with a differential diagnosis of Becker's muscular dystrophy. Supportive management including counselling and prednisolone tablet was commenced on outpatient basis, and he was scheduled for follow up appointment. However, the patient did not come back for follow up visit despite numerous phone call appeals.

## Discussion

DMD is a chronic disease with multi-organ involvement. It requires multidisciplinary and integrative management with the Paediatric Neurologist coordinating the team care. Since all the disciplines may not be available in a hospital, thus coordinating the care of these patients might be very difficult. This poses a lot challenge in the management of these patients.

The parents of EC were poor with a large family size (5 male children) and were without any financial support from extended family members. This was reflected in their difficulty in paying for laboratory investigations. The investigations done were paid by the managing team. This highlights the need for free or subsidized health services for children with this crippling genetic disease.

Muscle biopsy for dystrophin assay was not done because we lack the facility for immune-cytochemical staining of muscle tissue for presence/absence of dystrophin. However, with the recent advances in mutational analysis of blood, muscle biopsy is no longer the definitive means of confirming DMD [7]. We also lack the facility for making molecular genetic diagnosis.

Children with DMD often experience depression and social isolation because the physical limitation of their disease may prevent them from playing with their peers. This might account for why EC was withdrawn on examination. His school performance was poor and it might have been sequel to the number of missed school hours because of difficulty with ambulation and repeated falls, although intellectual impairment is a feature of the disease [3, 5–8].

Emotional burn-out of the parents was due to the child's worsening muscle weakness and poverty. This might explain why they refused coming for follow up visits despite numerous phone call appeals, even though patient follow-up is very poor in our environment. Clearly, DMD disrupts the emotional, psychological and spiritual fabric of the entire family [9].

## Conclusion

A clinical diagnosis of DMD can be made based on history, physical examination and creatinine kinase screen, while the precise diagnosis is made via molecular genetics [7, 8]. Therefore Paediatricians practicing in resource poor settings should not wait until dystropin assay is done on muscle tissue before a making a diagnosis of DMD to avoid late diagnosis. Creatinine kinase should be measured because it is simple to perform, rapid and cheap [10].

To address the issues in caring for children with DMD, we advocate for the provision of free health services, special schools and the establishment of DMD support groups for these special children.
